# Clinicians’ Emotional Reactions toward Patients with Depressive Symptoms in Mood Disorders: A Narrative Scoping Review of Empirical Research

**DOI:** 10.3390/ijerph192215403

**Published:** 2022-11-21

**Authors:** Alberto Stefana, Paolo Fusar-Poli, Cristina Gnisci, Eduard Vieta, Eric A. Youngstrom

**Affiliations:** 1 Department of Brain and Behavioral Sciences, University of Pavia, 27100 Pavia, Italy; 2 OASIS Service, South London and Maudsley NHS Foundation Trust, London SE5 8AZ, UK; 3 Early Psychosis: Interventions and Clinical-Detection (EPIC) Lab, Department of Psychosis Studies, Institute of Psychiatry, Psychology & Neuroscience, King’s College London, London WC2R 2LS, UK; 4 Riabilmente—Centro di Riabilitazione Monterotondo, Monterotondo, 00015 Roma, Italy; 5 Bipolar and Depressive Disorders Unit, University of Barcelona Hospital Clinic, IDIBAPS, CIBER-SAM, 08007 Barcelona, Catalonia, Spain; 6 Department of Psychology and Neuroscience, University of North Carolina at Chapel Hill, Chapel Hill, NC 27599, USA; 7 Helping Give Away Psychological Science (HGAPS.org), Chapel Hill, NC 27599, USA

**Keywords:** countertransference, emotional responses, in-session process, depressive symptoms, mood disorder

## Abstract

The purpose of this article is to narratively review the empirical literature on clinicians’ emotional, cognitive, and behavioral responses (i.e., countertransference) to depressive and other symptoms of patients with mood disorders. Therapist subjective responses (countertransference) can negatively affect both diagnostic and therapeutic processes, especially when they are not recognized and managed promptly. However, at the same time, countertransference recognition, processing, and management can help inform the diagnostic process and improve the therapy process and outcome. In the last couple of decades, the number of studies that empirically explore countertransference toward mood disordered patients, as well as its relationship with various characteristics of both patients and treatment, has increased. Current evidence suggests that patients with depression tend to elicit more positive feelings among clinicians than patients with other severe mental disorders such as borderline personality disorder or schizophrenia. Furthermore, it documents the existence of associations between patients’ severity of depressive symptoms and clinicians’ subjective reactions, although the results regarding which specific countertransference patterns are evoked in relation to the different phases of the treatment are not entirely consistent. Lastly, growing evidence suggests the presence of clinicians’ specific emotional reactions towards patients with suicidal ideation and behavior.

## 1. Introduction

### 1.1. Mood Disorders

Mood disorders are characterized by striking and persistent mood swings that result in notable psychological distress and/or behavioral impairment. They are classified according to the extent and severity of mood elevation, along a continuum ranging from unipolar depressive disorders to bipolar disorders [1,2]. Mood disorders often onset between adolescence and young adulthood [3] and affect more than 300 million people worldwide [4]. They frequently co-occur with other health conditions and substance misuse, and can cause cognitive impairment, social dysfunction, and poor quality of life [5]. In fact, unipolar depressive disorders are the third leading cause of years of lived with disability [4], while bipolar disorder is ranked among the top 20 causes of disability among all acute and chronic diseases and injuries worldwide [6]. Furthermore, people with a mood disorder are at high risk for chronic medical conditions [7,8,9] and suicide-up to 30 times higher than in the nonpsychiatric population [10]. All this comes at high costs for patients, families, and society.

Pharmacotherapy is the first treatment option selected by many patients, and particularly for severe depression or bipolar disorders [11], but growing evidence indicates that many patients still struggle to achieve good functional recovery [12,13]. The role of psychotherapies as adjunctive treatment is crucial in promoting positive function and producing behaviour and lifestyle change essential for relapse prevention and long-term maintenance [14,15]. Hence, psychotherapies are evolving towards evidence-based, personalized interventions [16].

Although notable progress has been made in our comprehension and management of mood disorders over the last decades, psychiatry is still far from being able to provide personalized and precise biological and psychosocial interventions for patients with affective disorders [17,18]. The treatment of these disorders remains, largely, a subjective clinical practice [19]. Implementing evidence-based assessment (EBA) and treatment for mood disorders is essential to reduce their burden at individual, societal, and public health levels. An assessment process is essential because it leads to a more accurate diagnosis [20,21], better treatment matching [22,23], increased patient engagement [24,25,26], and enhanced outcomes [27,28]. However, to be fully effective, EBA algorithms must be “humanized” because both diagnostic and therapeutic processes take place within the patient–clinician relationship [29,30,31], which can be defined quite generally as the feelings and attitudes that the clinician and the patient have toward one another, and the manner in which they are expressed [32], is a key aspect of both diagnostic and therapeutic processes [33,34,35]. The technical and relational parts of the therapeutic relationship are interlinked; and the relationship accounts for process and outcome variance in and of itself. Countertransference is a key element of the therapeutic relationship [36,37].

### 1.2. Working with Depressive Symptoms in Patients with Mood Disorders

Studies on emotional contagion and symptom transmission are consistent in showing that one person’s emotional and psychological state may significantly affect another’s, especially within a close relationship [38]. Meta-analytic findings indicate that depressive symptoms are “contagious” in the sense that they are likely to induce similar negative emotions in dyadic interactions [39] (see also [38]). Consistently with these findings, clinicians report that working with a patient with depression can also affect their own moods, and they must be careful not to fall too deep into a negative state [40]. Clinicians are generally poorly prepared to protect themselves from vicarious experiences while empathically coming close to the depths of the patient’s depressive state and trying to help them resolve it [41]. These negative reactions can gradually and subtly burden clinicians and deteriorate both their well-being and zeal for clinical work. In fact, evidence shows that depressed patients have a significant stressful impact on their psychotherapists [42] and that negative experiences with difficult patients represent an important risk factor for burnout [43]. Furthermore, a qualitative study exploring the in-session emotional reactions of psychotherapists who have personal histories of psychiatric hospitalization showed that these “wounded healers” experienced a range of emotional issues, including excessive degrees of identification with their patients [44]. Lastly, a study investigating the effects of patients with different diagnoses on therapists’ inner experiences revealed that patients with depression elicited the highest degree of depression in the therapists [45].

Patients with psychiatric symptoms, such as depression and suicidality, as well as challenging behaviors are sometimes considered “difficult patients” [46]. In addition to the mechanisms of symptom contagion described above, dealing with these patients can be complicated because of their helplessness and hopelessness. These can trigger strong negative emotions and thoughts, which in beginning clinicians can give rise to doubts about professional suitability and feelings of inadequacy, and in experienced clinicians could lead to cynicism and discouragement about treatment effectiveness [47,48,49].

The inner experiences of clinicians while working with their patients can negatively affect both diagnostic and therapeutic processes, especially when these experiences are ignored or not recognized and managed promptly [50]. Here, it should be noted that a not negligible number of clinicians tend to label their own inner experiences with patients as subjective and idiosyncratic, or even devalue them as unprofessional, interfering, and counterproductive [48,49]. By considering their own emotional reactions as interference in the diagnostic or therapeutic process, or even denying them, clinicians increase the risk of both developing burnout and “missing an important source of data that may directly or indirectly affect the (…) alliance and negatively influence treatment outcomes” [48] (p. xiii).

Maintaining a professional and clinically useful attitude while dealing with negative emotional reactions that are frequently experienced during a session is one of the greatest challenges for the clinician [50]. To help prepare clinicians and better help patients, we need to know more about clinician feelings and thoughts when meeting patients with depressive symptoms or disorders.

### 1.3. Countertransference and Therapist Subjective Reactions

In 1909, Sigmund Freud introduced the term countertransference (CT) to describe the emotional difficulties the psychiatrist and analyst Carl Gustav Jung had been encountering in the therapeutic relationship with one of his patients [51]. Freud conceptualized the therapist as a blank screen onto which the patient can project their own inner world, and CT as an obstacle to treatment that needed to be avoided or overcome. Building on this base, early psychoanalysts tended to see the therapist’s affective and behavioral responses to the patient just as an unconscious and neurotic response to the transference of the patient, or rather as a barrier aroused/elicited/induced within the therapist by the patient [52]. However, starting from the late 1940s, with the papers of Heinrich Racker and Paula Heimann [53], the idea of CT as a potential tool for understanding the patient’s inner world started to gradually become widely accepted, along with a more general recognition that the clinician’s identity and subjectivity are inseparable from being a person with emotions that are triggered within a bi-personal field. This view denoted a turn from a one-person view to a two-person view of the therapeutic situation. An important implication is the conceptualization of the clinician’s emotional responses to the patient as a complex and jointly created phenomenon that involves contributions from both members of the therapeutic couple [54].

From the early 1950s, CT began to expand beyond the boundaries of psychoanalysis, and the first attempts to empirically investigate the inner experiences of the clinician started [54,55,56,57]. However, empirical research progressed slowly during the first decades, mainly because of the extreme complexity of the phenomenon, the existence of various, often conflicting, definitions of it, the methodological issues in measuring it, and the strong reluctance of clinicians to be objects of research. Some reasons for the reticence include attribution of low or no clinical value to this phenomenon, the fear of being judged or negatively evaluated, and/or a mere shortage of time [58].

However, the publication of the third edition of the Diagnostic and Statistical Manual of Mental Disorders (DSM-III) [1], which introduced a multiaxial classification system, improved empirical research on the clinician’s inner experience and motivated the investigation of its possible association with patient diagnosis (particularly personality disorders) [59,60,61,62]. Consequently, research has been growing since 1980, accumulating substantial empirical evidence that indicates the pantheoretical status of CT and that its management is related to psychotherapy outcomes [63].

The trans-theoretical status of CT has been clearly demonstrated: the phenomenon is characteristic of interpersonal relationships in general and, therefore, occurs across any kind of psychotherapy and psychiatric sessions [64,65]. Growing evidence indicates that recognition, working through, and management of emotional responses towards the patient can help inform the diagnostic process [66,67]—for example, by giving information about the patient’s personality functioning [68] and interpersonal style—and improve therapy outcome [63,69]. Here, it must be underlined that it is not the emotional response in and of itself that is the mechanism for understanding what happens in the patient or in the relationship with them. It is rather the result of the continuous reflection/investigation/processing that the clinician makes of his/her own reactions. Additionally, the clinician should adopt both an idiographic and nomothetic perspective, maintaining tension between these two approaches [70,71].

### 1.4. Aims of the Present Review

Therapists’ subjective experiences are affected by the dyadic interaction of therapy. Their experiences are related to aspects of therapeutic process and outcome and to therapist self-perceptions of efficacy or burn out. The literature also is clear that these experiences are not limited to psychodynamic modalities (where they are explicitly conceptualized as “countertransference”), but rather appear to be part of a general core role for affective experience in dyadic human interactions. Patient diagnoses or mood states might also affect the dyad and induce predictable and often congruent mood states in the therapist. Therefore, the goal of the present project was to conduct a scoping review of the literature on countertransference and therapist subjective reaction, with a focus on mood disorders, while searching across therapeutic modalities. Mood disorders provide a form of “natural experiment” with varying mood presentations, extending from severe depression to euthymia and then hypomania, agitated depression, mania, and mixed presentations. Mood disorders also have high probability of progress and episode remission, as well as recurrence, and a high risk of self-injurious behavior, introducing a wide range of clinical presentations both within and between patients.

## 2. Methods

The databases CINAHL, PsycINFO, PubMed, and Scopus were searched by abstract each database’s date of inception to October 2022. In addition, reference lists and foreword citation searches were performed. The literature search included the terms (“depression” OR “depressive” OR “mood disorder” OR “bipolar disorder”) AND (“countertransference” OR “emotional reaction” OR “emotional response” OR “subjective experience”) AND (“clinician” OR “therapist” OR “psychotherapist” OR “psychologist” OR “psychiatrist”) and was limited to English-language peer-reviewed journal articles.

## 3. Results

**Figure 1:**
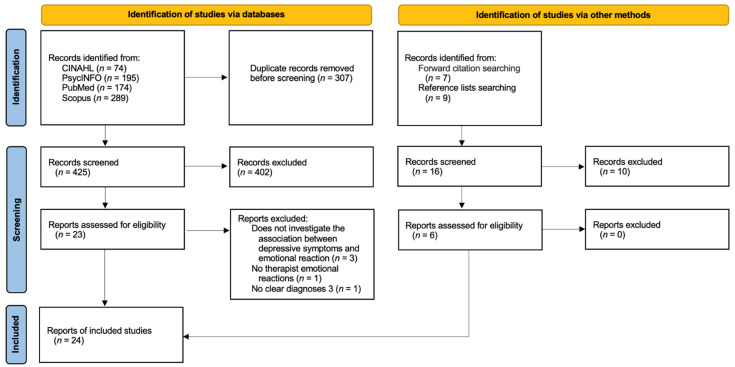
PRISMA diagram of study selection process.

The initial search retrieved 425 journal articles, 16 additional articles were captured via reverse search strategies (Figure 1). Of these, 24 articles were included in the present review. Table 1 lists and describes the measures for the assessment of clinicians’ reactions toward the patient used in the studies included in this review.

### 3.1. Comparison between/among Different Diagnostic Groups

Brody and Faber [45] explored the effect of patients’ diagnoses and psychologists’ clinical experience on CT. In their study, a total of 336 participants (71 students in clinical psychology programs, 39 interns/Ph.D. candidates, and 217 licensed psychologists) completed the Experience and Attitude Scale [45] and the Vignettes Rating Scale [45] to rate attitudes toward their emotional reaction to the reading of vignettes describing patients diagnosed with major depression, borderline personality disorder, or schizophrenia. The results showed that compared to patients with borderline disorder or schizophrenia, patients with depression evoked a predominance of positive CT reactions (i.e., empathy, nurturing feelings), as well as the highest degree of depression in the therapist. On the other hand, patients with borderline personality disorder elicited mainly negative CT feelings (i.e., anger and irritation), while patients with schizophrenia elicited a mixture of anxiety, hopelessness, and frustration. Regarding the level of clinical experience of the raters, compared to licensed psychologists, students and interns were more likely to regret saying things to the patient and to feel their own emotions as too strong, too frequent, and needing to be defended against.

McIntyre and Schwartz [72] investigated the personal perceptions and emotional reactions of 155 licensed psychotherapists toward a patient with either major depression or borderline personality disorder. Therapists rated both the Impact Message Inventory [73] and the Stress Appraisal Scale [74] after having listened to a diagnostic interview session of a patient prototypical of their diagnosis. Therapists were more likely to feel that patients with major depression elicited reactions such as agreeability, care, importance, nurturance, submissiveness, and succorance, while patients with borderline personality disorder elicited reactions such as competition, detachment, and mistrust. Interestingly, the authors found that as the years of experience of the therapists increased, the degree of CT reactions decreased.

Pallagrosi et al. [75] investigated the relationship between diagnostic groups and clinicians’ subjective experiences. The study involved 24 psychiatrists, 11 senior psychiatry residents, and 422 patients attending inpatient or outpatient psychiatric units. The patients were diagnosed with unipolar depression and anxiety disorder (31%), bipolar I disorder (14%), schizophrenia (28%), and cluster B personality disorder (27%). The clinicians were asked to complete the Assessment of Clinician’s Subjective Experience (ACSE) [76] questionnaire when they saw a new patient. The results indicated that patients with unipolar depression and anxiety elicited less intense reactions than all other groups on the ACSE scales difficulty in attunement, disconfirmation, and impotence. Compared to those with unipolar depression and anxiety, patients with a bipolar manic or mixed episode elicited higher scores on tension, impotence, difficulty in attunement, and disconfirmation. Furthermore, patients with unipolar depression evoked significantly higher engagement than patients with a cluster B personality disorder.

Putrino et al. [77] interviewed 43 clinical psychologists–psychotherapists with different theoretical orientations to investigate their perceptions of their CT reactions toward patients diagnosed with major depressive disorder or borderline personality disorder. Most therapists reported negative feelings such as annoyance, helplessness, and frustration, as well as physiological reactions such as mental exhaustion, drowsiness, and headache when treating these patients. The same sample of therapists reported a similar level of negative feelings related to working with patients with borderline personality disorder, but with a range of physiological reactions that also included muscle tension and increased heart rate.

### 3.2. Clinician’s Response to Patient’s Characteristics

Several studies have shown significant associations between patient characteristics (such as severity of symptoms, psychological functioning, and personality pathology) and clinician emotional responses.

Røssberg et al. [78] investigated the relationship between patients’ self-reported symptoms and therapists’ CT. Their sample consisted of 42 patients, most of whom had a diagnosis of mood disorder (81%) and comorbid personality disorder (100%), and 11 therapists. The latter were asked to complete the Feeling Word Checklist 58 (FWC-58) [79] during their last conversation of an 18-week (4 days a week) treatment program consisting of a combination of psychodynamic and cognitive behavioral-oriented group therapies. The results indicated that having a higher number of depressive symptoms at the end of the treatment was associated with therapists having less confidence and stronger feelings of being inadequate. No associations were found between CT feelings and the patient’s symptoms at the beginning of treatment. More generally, the authors found that higher levels of psychiatric symptoms at the end of the treatment were negatively associated with therapists’ feelings of being important and confident and positively associated with feelings of being bored, on guard, overwhelmed and inadequate.

These results have been confirmed by another study that explored the relationship between the FWC-58 factors and a variety of patient characteristics. Dahl et al. [80] asked six experienced psychodynamic psychotherapists to rate their emotional reactions to patients over the psychotherapy period. Seventy-five patients, 45% of whom met the criteria for DSM-III depressive disorders, were included in the analysis. A mean of 32 questionnaires were filled out for each patient. The results did not show correlations between CT feelings and self-reported levels of depression before treatment. However, significant negative associations were found between the number of personality disorder criteria and Confident and Disengaged CT.

Datz et al. [81] explored the interaction structure between CT reactions of therapists and the emotional functioning of patients with major depression as the primary diagnosis. Audio-recorded sessions (*N* = 639) from the beginning, the middle phase, and the end of psychotherapeutic treatments (i.e., psychoanalysis, psychodynamic psychotherapy, and cognitive behavior therapy) of 100 patients were rated by external blind, trained raters using the German translations of the Psychotherapy Process Q-set (PQS) [82] and the Therapist Response Questionnaire (TRQ) [83]. The raters were fourth-year medical students without any psychotherapeutic training. Principal component analysis showed that the behavior of the therapists (PQS items) formed two major dimensions they named “supportive behavior” and “disrespectful behavior,” while their emotional reactions (TRQ scale) were characterized by five dimensions: hostile feelings, positive feelings, disengaged feelings, overwhelmed feelings, encroaching feelings. Based on these results, the authors hypothesized that the emotional reaction of the therapist and the behavior possibly motivated by or associated with these emotions are two very different parameters. Furthermore, the results of the study indicated that the middle and final sessions reveal significant associations between the positive affect of the patient and the positive (satisfying) CT feelings.

The same research group also examined whether part of the variance in CT responses toward patients can be attributable specifically to (a) the person of the patients and (b) their clinically relevant features (including double depression, severity of symptoms, and comorbid personality disorder) [94]. Six fourth-year medical students (external raters) listened to a sample of 605 audiotaped psychotherapy sessions of 81 patients diagnosed with major depression treated by 19 experienced therapists of different theoretical orientations (psychoanalytic, psychodynamic, and cognitive-behavioral) and rated their own CT reactions using the German version of the TRQ. None of the raters had undergone any psychotherapeutic training. The authors found that the average relative variance due to patients across observer-rated CT reactions was 9%. It was significant on five of seven TRQ scales: helpless/inadequate (16%), hostile/mistreated (12%), disengaged (12%), positive/satisfying (9%) and parental/protective (5%) reactions. The variance was not different from zero on the remaining two scales. CT reactions toward patients treated with psychoanalytic psychotherapy were less positive/satisfactory and involved more difficult feelings and thoughts than reactions in patients treated with psychodynamic therapy or cognitive behavior therapy. Furthermore, the results suggest that patients with more severe depressive symptoms at the beginning of therapeutic treatment evoked more helpless/inadequate and less positive/satisfying reactions over the course of therapy. No evidence was found on the effect of comorbid personality disorder on CT reactions in addition to the effects of depressive symptoms.

Picardi et al. [95] explored the relationship between the psychopathological dimensions of patients and the reactions of clinicians. In a large sample (45 clinicians and 783 patients—one-third of whom had a mood disorder) [75], the authors found that more severe depressive symptoms were associated with higher clinicians’ feelings of impotence (including feelings of being drained, desolation, emptiness, frustration, helplessness, and loneliness). Furthermore, younger clinicians were significantly more likely to experience feelings of impotence.

Moukaddam et al. [96] investigated the relationship between CT and diagnosis and severity of symptoms in patients who profess religious faith vs. not. Forty clinicians (24 psychiatrists and 16 psychotherapists) were exposed to three videotaped interviews with standardized patients (actresses) who showed similar degrees of depression but professed different religious beliefs. After exposure to the vignettes, the clinicians rated the severity of depression of the patients and completed the TRQ. The results showed that patients without religion were rated as less depressed than those with religion, seemingly contradicting part of the literature on religiosity as a support/coping resource/protective factor [97,98]. Furthermore, the psychiatrists’ ratings presented a positive linear relationship between depressive symptomatology scores and protective CT, a negative linear relationship between depressive symptomatology scores and overwhelmed CT, and a nonlinear but significant relationship between depressive symptomatology and positive and disengaged CT reactions. Regarding the therapists, a positive linear relationship was found between depressive symptomatology scores and protective and positive CT reactions, as well as a negative linear relationship between depressive symptoms and helpless and disengaged CT.

The following three studies specifically investigated the associations between the patient’s personality and the CT of the therapist.

Betan et al. [88] examined associations between CT reactions and the personality pathology of patients using a sample of 181 patients whose most common diagnoses were major depressive disorder (49%) and dysthymic disorder (38%), respectively. Therapists (*N* = 181) were asked to rate the TRQ by trying to describe the way they felt with a specific patient over the course of the entire (ongoing) psychotherapeutic treatment. Due to the high comorbidity of the axis II disorders, the authors analyzed the data at the personality disorder cluster level by summing the number of DSM-IV symptoms endorsed for each personality disorder included in each cluster. The results showed that cluster A personality disorder was significantly associated with criticized/mistreated reactions. Cluster B was positively associated with overwhelmed/disorganized, helpless/inadequate, special/overinvolved, sexualized, disengaged, criticized/mistreated, while it was negatively associated with positive responses towards the patient. Lastly, cluster C was directly associated with parental/protective responses.

Using the Psychodynamic Diagnostic Manual—Second Edition (PDM-2) [34] classification of personality disorders, Genova and Gazzillo [99] asked 232 clinicians to assess the personality syndromes of one of their patients (with heterogeneous diagnoses), as well as their own emotional reactions toward these patients using the Italian version of the TRQ [89]. The results showed that the depressive/hypomanic cluster of personality syndromes was predicted by parental and disengaged CT responses. In terms of specific personality syndromes and their subtypes, the findings indicated that the types of depressive personality were predicted only by a disengaged CT response, while the introjective subtypes were also predicted only by a disengaged CT reaction. No significant association was found between the anaclitic subtypes and TRQ factors. On the other hand, hypomanic personality styles (in which driven, obligatory optimism and energy defend against underlying depressive affect) were associated with sexualized and helpless CT reactions.

Hennissen et al. [100] explored the responses of therapists to depressed patients with dependent (anaclitic) and self-critical (introjective) personality styles, as well as the relationship between these responses and patient (i) severity of symptoms and (ii) therapeutic change. Data collection was part of a randomized controlled trial comparing cognitive behavior therapy and short-term psychodynamic therapy in the treatment of outpatients with major depressive disorder. Eight therapists (4 for each treatment group) completed the TRQ for 34 introjective and 50 anaclitic depressed patients at five time points over the course of short-term (20 sessions) therapies. The results showed that the therapists experienced stronger parental/protective responses to dependent (anaclitic) patients than to the self-critical (introjective) ones. No other significant associations were found between the personality styles of depressed patients and other dimensions of the TRQ. Furthermore, the findings indicated that cognitive behavior therapists reported stronger reactions than dynamic therapists. No significant associations were found between the severity of the symptoms of the patients at the beginning of treatment and the overall intensity of the initial cognitive and emotional responses of the therapists, but the latter experienced stronger responses when the patients did not make therapeutic progress.

### 3.3. Countertransference and Psychotherapy Elements, Process, and Outcome

Dahl et al. [101] also explored the impact of CT on the therapeutic process. Ninety-nine outpatients who sought psychotherapy for mental problems, including depression (64%) and personality disorders (45%), were randomly assigned to one of two one-year treatments: dynamic psychotherapy with vs. without transference work. Therapists’ CT feelings were assessed using the FWC-58 after each session. The outcome was assessed before treatment, mid-treatment, post-treatment, one year, and three years after treatment. The results indicated that disengaged CT feelings, even in small amounts, were associated with negative long-term effects of the transference interpretation work. The strengths of this negative relationship increased significantly with lower levels of patient’s quality of past and present interpersonal relationships.

Tanzilli et al. [102] examined the relationships between CT and therapeutic alliance, as well as between these elements of therapeutic relationships and the outcomes of short-term psychodynamic psychotherapy. The sample consisted of 20 psychotherapists of different theoretical orientations and 32 patients with mood- or anxiety-disordered and relational problems. Therapists were asked to complete the ACSE after the first interaction with patients and the TRQ (along with the therapist version of the Working Alliance Inventory [91]) at the end of the sixth therapy session. The results showed that strong and more negative CT reactions were associated with a lower quality of the therapeutic alliance. More specifically, the alliance was positively correlated with the ACSE engagement scale and the TRQ positive/satisfying pattern. At the same time, it was negatively associated with ACSE difficulty in attunement, disconfirmation, and tension, as well as with TRQ criticized/devalued, disengaged, helpless/inadequate, hostile/angry, and overwhelmed/disorganized patterns. Regarding the relations with the outcome of the therapy, the improvement in symptoms was negatively associated with ACSE attunement and TRQ disengaged, hostile/angry, and helpless/inadequate patterns. No associations were found between patients’ improvement in psychiatric symptoms and positive CT (i.e., ACSE engagement and TRQ positive/satisfying pattern).

Falkenström & Holmqvist [103] investigated the effect of therapist feelings on patient depressive symptoms in Brief Relational Therapy (BRT) [104] compared to Interpersonal Psychotherapy (IPT) [105]. Fourteen therapists (psychologists or social workers) were involved in the study; each of them was to treat six patients (three using BRT and three using IPT) who met the diagnostic criteria for major depressive disorder. Therapists were asked to complete the FWC-24 [86] immediately after each session. In sessions in which the therapist felt more engaged during the session, the therapeutic alliance was better, and the patient experienced fewer or improved depressive symptoms in the next session. Similarly, the more the therapist felt engaged, the more the patient also tended to feel engaged (especially if they were in the IPT group), and this patient’s greater engagement predicted increased depressive symptoms in the next session. However, if the patient’s feelings of engagement and the alliance were not related to the therapist’s feelings of engagement, the patient felt less symptoms in the following session. Furthermore, when the therapist experienced more relaxed feelings, the alliance would be better in that session, and the patient would feel decreased depressive symptoms in the following session—this happened in both the BRT and IPT groups.

Brøsholen et al. [85] also recently investigated the relationship between the CT and alliance (from both patient and therapist perspectives) using a randomized controlled trial in which eleven child and adolescent psychotherapists treated patients (*N* = 68) aged 16–18 years over a period of 28 psychodynamically oriented sessions. The therapists rated their CT reactions on the Feeling-Word Checklist-28 (FWC-28) after sessions 3, 12, 20, and 28. The FWC-28 is an expanded version of the FWC-24, specifically designed by Brøsholen et al. to assess CT responses experienced by therapists when treating adolescent patients. All four FWC-28 subscales showed a significant correlation with the therapist version of the Working Alliance Inventory [106]. More specifically, the alliance was negatively correlated with the inadequate and disengaged CT subscales, and positively correlated with confident and motherly CT subscales. Furthermore, there was a significant positive correlation between the alliance as rated by the patient and the confident CT subscale.

Roubal and Rihacek [41] conducted individual and focus group interviews with 30 therapists to investigate their experiential process during a session with a currently depressed patient. The qualitative analysis revealed that the experience of the therapists can be conceptualized as an experiential oscillation between getting closer to the depressive experience of the patient and moving away from it. This process seems to be composed of six phases that develop over the course of the therapy session: (1) sharing the depressive experience, (2) turning to oneself, (3) striving for symptom change, (4) distancing from the depressive experience, (5) turning to a client, and (6) focus on the relationship. It must be noted that this general sequence can happen once during the session, can repeat itself more than once during one session, or can develop only parts of its complete trajectory. Furthermore, three “process variants” of the depression co-experiencing trajectory were identified. According to the authors, the resultant theoretical model can coherently interconnect different CT reactions to a depressive patient within a process model that allows tracking changes in therapists’ emotional experiences, naming the relations between them, and linking them to in-session microprocesses.

Hennissen et al. [107] studied the reactions of therapists towards (quasi) prototypical dependent (anaclitic) depressed patients through a qualitative methodology. The verbatim transcriptions of seven supervision sessions of psychodynamic therapists discussing their clinical work were analyzed. Thematic analysis revealed four recurrent themes: (a) empathy, compassion, and support; (b) anxiety, feeling overwhelmed, and protection; (c) frustration, irritation, and confrontation; and (d) inadequacy, incompetence, and fatalism. The theme (a) describes positive feelings such as benign affection, empathy, and sympathy, as well as attentiveness to the material brought about by the patient and supportive interventions. These reactions were experienced primarily in the context of a good alliance, perceived psychological/physical vulnerability and suffering of the patient, and/or initial discomfort of the patient within the therapeutic setting. The theme (b) contains feelings of alarm, fear, responsibility, and overwhelm, as well as protective and containing interventions in situations where the therapists experienced the patient as at risk of psychological disintegration. Furthermore, it contains feelings of anxiety in situations where a patient has experienced strong interpersonal dependency. The theme (c) describes feelings of irritation, anger, frustration, and resentment, as well as hostility and loss of patience. These responses were experienced when patients were perceived to lack motivation, resistant to treatment, or have a strong interpersonal appeal. The theme (d) contains feelings of self-doubt, inadequacy, and incompetence, as well as helplessness, hopelessness, and fatalism. These feelings and reactions were related to clinical situations where the therapist was unable to overcome the resistance of the patient or where the last was perceived as a risk of psychological disintegration.

### 3.4. Clinicians’ Responses to Suicidal Patients

There is a growing literature exploring the reactions of healthcare professionals toward suicidal patients. Michaud et al. [108] conducted a systematic review of quantitative research on this topic and identified ten studies that overall showed evidence for specific and adverse clinician cognitive and emotional reactions (including anxiety, distress, overwhelming, disinterest, rejection, and helplessness) toward suicidal patients. Furthermore, they found that clinicians’ responses were prospectively associated with suicidal ideation and behavior, but the meaning of this association was unclear. Another finding was that clinicians’ characteristics such as gender, personality traits, and professional background influenced their own reactions.

Among the studies included in Michaud et al.’s [108] review, three are particularly interesting for our discussion because they included patients with a diagnosis of mood disorder.

Colson et al. [59] investigated the CT reactions of hospital personnel to difficult-to-treat psychiatric hospital patients diagnosed with personality disorder (*n* = 56), schizophrenia (*n* = 32), affective disorder (*n* = 20), and other psychosis (*n* = 19). The members of eleven treatment teams (each composed of a psychiatrist, an activity therapist, a nurse, and a social worker) rated a 16-item feelings checklist to each patient. Suicidal-depressed behavior was not consistently associated with emotional reactions in both psychiatrists and activity therapists, but it tended to elicit positive interest and protectiveness and a general lack of dysphoric feelings among nurses and social workers. On the other hand, personality pathology was strongly associated with anger, withdrawn psychoticism was most strongly associated with helplessness, whereas violence-agitation elicited helplessness within psychiatrists, anger within activity therapists, and fearfulness within nurses and social workers (the latter also experienced positive engagement). One countertransference issue suggested by these findings may be that hospital personnel unconsciously prefer those patients whose aggression is self-directed, thereby allowing clinicians to be in the socially more desirable stance of emotionally positive/protective engagement with these patients.

Markin et al. [109] examined how transference and CT were related to one another and to aspects of the quality of the therapy session. The study was based on the ratings of 115 videotaped psychotherapy sessions related to 44 patients participating in a randomized clinical trial on supportive-expressive therapy for depression. Therapies were carried out by four psychodynamic therapists with at least 10 years of clinical experience. Five raters (upper-level students in a master’s program in counseling or psychology with 1 to 2 years of clinical experience) rated measures of transference, CT (using the Countertransference Behavior Inventory [84]), therapist’s emotional expression (using the Therapist Positive Feeling Index [87]), and session quality immediately after watching a videotaped session. In most cases, three sessions (i.e., at the beginning, middle and end therapy) were rated for each patient. In contrast to the hypotheses of the authors, positive or negative CTs were not predicted by the quality (positive vs. negative) of the transference, nor did they vary over time. However, greater negative transference significantly predicted more negative therapist emotional expression at every session. Positive CT and positive mood predicted a smooth (i.e., comfortable and pleasant) but superficial session, with positive transference serving as a moderator.

Barzilay et al. [92] explored the relationship between clinicians’ emotional responses and patients’ suicide outcome measures. Forty-eight mental health trainees and professionals (psychiatrists, psychiatry residents, attending psychiatrists, attending psychologists, and social workers) were asked to rate the Therapist Response Questionnaire—Suicide Form (TRQ-SF) [90] and other measures after their first clinic meeting with adult psychiatric outpatients (*N* = 346). Patient symptom severity and suicidal outcomes were assessed both at intake and at one month of follow-up. 45% and 13% of the patients had, respectively, depressive disorder and bipolar disorder as primary diagnosis. The TRQ-SF total score and three subscales (i.e., affiliation, distress, and hope) were associated in both concurrent and predictive ways with the severity of the patient’s depression, the judgment of the clinicians on the risk of suicide of the patient, and the suicidal outcomes, but not with the global severity of psychopathology.

In addition to the studies included in the systematic review mentioned above, Michaud et al. [93] evaluated the impact of suicidal ideations and self-harm on CT. Thirty mental health professionals (physicians, psychologists, nurses, and social workers) used the TRQ-SF and the Feeling-Word-Checklist 30 (FWC-30) [110] to rate their CT with 321 patients visited in an emergency ward (82%) or in a specialized outpatient clinic for depressive disorders (18%). Of these patients, 54% and 7% had, respectively, a diagnosis of depressive and bipolar disorders. Furthermore, 38% of the entire sample had a diagnosis of personality disorder. The result indicated that suicidal ideation levels and the presence of personality disorder were independent predictors of the TRQ-SF total score and of several sub-scores. Common CT reactions included tension, lack of self-confidence, and feeling of being tied. Reactions specifically associated with suicidal ideation included distress, confusion, lack of hope, and the sense that the patient’s life was little worth. CT with respect to patients with personality disorders included anger, disappointment, devaluation, and low affiliation with the patient.

## 4. Discussion

Therapist subjective reactions (often conceptualized as CT) are a ubiquitous and potentially useful phenomenon for mental health clinicians in various diagnostic and treatment situations and settings [54,63,111,112,113]. It is the result of a complex combination of the intrapsychic interactions of the clinician and the interpersonal dynamics between the clinician and the patient, the latter being intrinsically linked to the characteristics of the patient. The aim of the present paper was to review the literature on therapist subjective reactions (CT) regardless of therapeutic modality, while concentrating on patients with mood disorders as a way of focusing on how variations in patient mood or clinical features might affect the therapist’s subjective experiences. Unlike more chronic conditions, the episodic nature of mood disorders introduces the possibility of both progress and setback in the therapeutic work, and when considering bipolar disorders as well as unipolar depression, there is a broad range of affect associated with the clinical presentation.

The present review shows that patients with depression tend to elicit more positive feelings among clinicians than patients with other severe mental disorders such as borderline personality disorder or schizophrenia. In addition, it documents the existence of associations between the severity of the depressive symptoms of patients and the CT responses of the clinicians, although the results regarding the specific CT patterns evoked in relation to the different phases of treatment are not entirely consistent. This could be due to methodological differences, particularly the use of different measures to assess both the CT and the clinical features of patients.

Although the number of empirical studies that empirically explore CT toward patients with depressive symptoms or disorders, as well as its relationship with various patients’ and treatment characteristics, have been slowly increased in recent years, there is still a relative dearth of empirical evidence in this area. However, the available evidence has the potential to increase clinicians’ awareness of common and therefore predictable CT general reactions toward depressed patients. More research is needed to help clinicians understand how to use specific CT reactions to enhance their clinical work with this specific population. It is vital that mental health clinicians become more aware of their own emotional reactions and their impact on both the patients and the clinician-patient relationship.

Although the findings reported above are significant, there are some important limitations or problematics to be aware of. First, the studies differed even more with respect to the depressive states of the patients involved. Consequently, it is not possible to identify any potential associations between distinct depressive clinical pictures and specific clinicians’ emotional responses, and whether and how they vary within different settings (e.g., psychiatric interview, psychotherapy session, etc.). Second, despite the strong evidence on the impact of clinician’s interpersonal functioning and skills on treatment process and outcomes [114,115], there is a dearth of data on the broader spectrum of variables related to the person of the clinician and their possible moderating/mediating role in the elicitation of his/her own in-session emotional reactions while working with a patient. Third, data sets usually only include one or very few patients per clinician, this is not ideal for investigating clinicians’ reactions, as it does not allow statistical modeling of the possible therapist effect [116] on process and outcome. Fourth, almost all studies measure CT using self-report questionnaires, which may have important limits related to insight and objectivity. However, a body of literature supports the use of clinician-rating scales for countertransference [36,117,118]. Furthermore, clinicians’ self-reported emotional responses make perfect sense when considering the clinical importance of evaluating the subjective experience of each of the member of the therapeutic dyads [119]. Fifth, the cross-sectional design of most studies does not allow one to assess whether and how CT and its relationship with both patient characteristics and treatment outcome change over the different time/phases of the diagnostic or therapeutic process. Longitudinal assessment is the only way to determine with certainty whether and how the CT is an acute emotional reaction triggered by the characteristics of the patient, or a chronic emotional reaction provoked by the clinician’s personal difficulties, unresolved issues, or simply an emotionally bad day. Lastly, although strong evidence indicates the existence of a “contagiousness” of depression within a close relationship [38]—or rather the transmission of low mood, anhedonia, pessimism, etc. from a depressed person to a close one—research exploring this mechanism within the therapeutic relationships is still limited. It is presumable that the risk of such emotional contagion may be greater for clinicians treating severely depressed patients within an intense and prolonged relationship like the psychotherapeutic one.

A key future direction in this area of research should be to explore more fully the relationship between CT patterns and other key elements of the therapeutic relationship, such as the therapeutic alliance and the real relationship (see [37,120]), as well as to explore whether and how CT reactions and CT management influence the treatment process and outcome. Furthermore, it would be valuable to triangulate on both CT patterns and patients’ mood states and symptoms by using multi-informant and multi-method approaches to increase the reliability of the results. Regarding the patient diagnosis, empirical research is needed to investigate CT responses experienced by clinicians involved in the care of patients with bipolar disorder, even—or especially—during the acute phases of the illness [121,122,123]. Finally, future studies should longitudinally investigate the specific weight of the interlocking elements of the clinician’s reaction considering the personal and interpersonal characteristics of both clinicians and patients to better and deepen their understanding of what is inside CT, and of its diagnostic and/or therapeutic usefulness. Among the characteristics of clinicians, particularly important is the professional role (such as psychologist, psychiatrist, psychiatric nurse, social worker, etc.) because several studies showed that specific dimensions of patient psychopathology evoke different emotional reactions among different professional roles [124,125].

## 5. Conclusions

The purpose of this article was to provide an overview and summary of existing research on the relationship between patients’ depressive symptoms/mood and clinicians’ CT reactions. Almost four decades of empirical research indicate that patients with depressive disorders evoke more positive CT responses among mental health professionals than patients with other severe mental conditions such as schizophrenia or borderline personality disorder, and that the patients’ severity of the depressive symptoms is commonly associated with the clinicians’ CT responses (although the results regarding the specific CT patterns are not entirely consistent). Our findings overall suggest that the CT (and likely also its management) may be important for promoting adherence to diagnostic and treatment processes, which in turn would support positive therapeutic change. Given some inconsistent findings and methodological limitations, however, the relationship between CT and depressive symptoms/disorders and how it influences both the other elements of the patient-clinician relationship (e.g., alliance) and the patient outcomes in different settings and phases of treatment need further investigation. Since only a few process/outcome research studies include a CT measure, it would be useful to routinely include a psychometrically sound instrument in assessment batteries to eventually enable meta-analyses.

## Figures and Tables

**Table 1:** Measures used for the assessment of clinicians’ reactions toward the patient.

Measure Name	Description	Items/Factors/Subscales
Psychotherapy Process Q-set (PQS) Jones [82]	Q sort 100 items It is applied to recorded single session. Clinical judges sort items into nine categories from 1 (least characteristic) to 9 (most characteristic).	The items are divided into three categories that describe or attempt to capture the following areas: - Patient attitude and behavior or experience; - Therapist’s actions and attitudes; - Nature of the interaction in the therapeutic dyad or the emotional atmosphere of the encounter. Datz et al. [81] run a principal content analysis including the 29 items of the PQS Therapist’s actions and attitudes category and all the 79 items of the Therapist Response Questionnaire [83] and found that the PQS items loaded on two independent factors: - *Supportive behavior* - *Disrespectful behavior*
Countertransference Behavior Inventory (CBI) Friedman & Gelso [84]	Observational rating 21-items Designed for supervisors to assess perceived countertransference behavior during therapy sessions, rating reactions to a particular patient in a given therapy session. 5-point Likert scale ranging from 1 (to little or no extent) to 5 (to a great extent).	Exploratory factor analysis identified two factors: - *Negative countertransference* describes inappropriate therapist’s behaviors that are inappropriately disapproving of patient or not affirming in some way. - *Positive countertransference* includes therapist behaviors that appeared to be friendly or overly supportive. A *total score* is calculated sums the two subscales to estimate total amount of countertransference.
Assessment of Clinician’s Subjective Experience (ACSE) Pallagrosi et al. [76]	Clinician self-report 46 items measuring subjective experience during clinical interaction with patients. Each item refers to a specific phase of the visit: “at the beginning”, “in the course of” or “at the end”. 5-point Likert scale ranging from 0 (not at all/never) to 4 (extremely/always).	Principal component analysis identified five subscales. - *Tension* includes physical tension and clumsiness, reduced spontaneity, and feelings of nervousness, worry, and alarm. - *Difficulty in attunement* describes difficulty in establishing emotional contact, being empathic, understanding the patient’s experience and communicating with the patient. - *Engagement* describes the degree of involvement of the clinician in the patient, such as feelings of boredom, lack of attention, indifference, detachment, and, conversely, desire to take care of the patient and feelings of deep involvement in the therapeutic relationship, emotional closeness and tenderness. - *Disconfirmation* describes a failure to establish an authentic relationship with the patient, and the feelings of being manipulated, rejected, criticized or devalued by the patient. - *Impotence* indicates feelings of frustration, helplessness, desolation, loneliness, emptiness, and being drained.
Experience and Attitude Scale (EAS) Brody & Faber [45]	Clinician self-report 25 items Assesses the overall experience in psychotherapy (15 items) and attitudes towards their own emotional reactions in therapy (10 items). Clinicians rate the intensity or frequency of their reactions. 5-point Likert scale ranging from 1 (not at all/hardly ever) to 5 (very much so/very often).	The authors used only the subscale on the therapist’s attitudes toward their emotional reactions. Items were not summed, analyzing all 10 items separately. The aspects assessed by the items are as follows: - *Use feelings to understand* - *Act as blank screen* - *Regret saying things* - *Regret not saying things* - *Emotions too strong* - *Emotions too frequent* - *Emotions interfere* - *Defend against emotions* - *Need more personal therapy* - *Need more experience*
Feeling Word Checklist (FWC) Røssberg et al. [79]	Clinician self-report 58 items Therapists rate degree they have experienced different feelings toward the patient. 5-point Likert scale ranging from 0 (nothing) to 4 (very much). Note that some shorter versions (28- and 24-item versions) use a 4-point Likert scale ranging from 0 (nothing/not at all) to 3 (very much).	Principal component analysis identified four subscales: - *Confident* subscale includes the following feeling states: total control, clever, overview, attentive, receptive, confident, helpful, happy, enthusiastic, calm, and objective. - *Inadequate* subscale includes the following feeling states: inadequate, anxious, threatened, stupid, distressed, insecure, helpless, overwhelmed, cautious, rejected, disliked, embarrassed. - *Parental* subscale includes the following feeling states: motherly, affectionate, dominate, and important. - *Disengaged* subscale includes the following feeling states: tired of, sleepy, indifferent, aloof. Principal component analysis of both the 28-item [85] and the 24-item [86] versions identified the same dimensions, with the exception of the original Parental subscale changing into: - *Motherly* (on 28 item version)—feelings of being affectionate, warm, important, and in touch with the patient; - *Pragmatic* (on 24 item version)—two feeling states: prudent, neutral.
Impact Message Inventory (IMI) Kiesler [73]	Clinician self-report 90 items Measures distinctive internal reactions (referred to as impact messages) that the therapist experiences to the full range of interpersonal behaviors indexed along the circumference of the interpersonal circle. 4-point Likert scale ranging from 1 (not at all) to 4 (very similar).	Items are clustered into four subscales: - *Dominant* subscale describes feeling exhibitionistic, dominant, or competitive. - *Hostile*—feeling hostile, mistrusting, or detached. - *Submissive*—feeling abasive, submissive, or succorant. - *Friendly*—feeling agreeable, nurturant, or affiliative.
Stress Appraisal Scale (SAS) Carpenter & Suhr [74]	Clinician self-report 36 items Investigates the therapist’s feelings about entering a therapeutic relationship with a patient listened to in an audiotaped interview. 4-point Likert scale ranging from 1 (very untrue of me) to 4 (very true of me) by rating how accurate each statement is of the therapist’s reactions.	Items are grouped into four subscales, which in turn are clustered into three primary categories. - *Salience:* - Caring (concern about one’s performance) - Consequences (recognition that the outcome may have important consequences) - *Difficulty:* - Demands (beliefs that the stressor is significant) - Perception (feelings of anxiety) - *Secondary Appraisal:* - Skill (evaluation of one’s ability to cope with this individual) - Success (prediction of successfully coping with the expected stressor)
Therapist Positive Feeling Index (TPFI) Stiles [87]	Clinician self-report 6 items The TPFI is part of the Session Evaluation Questionnaire. It assesses the emotional expression of the therapist. The original version of this subscale begins with the stem ‘‘Right now I feel,’’ but was changed in the included study to, ‘‘To what extent did the therapist express feelings of __ during the session.’’ This stem is followed by the five bipolar adjectives that are rated on a continuum from 1 to 7 by a coder.	A total score is calculated using the mean rating on the following bipolar adjectives. - *Happy-Sad* - *Definite-Uncertain* - *Angry-Pleased* - *Friendly-Unfriendly* - *Confident-Afraid*
Therapist Response Questionnaire (TRQ) Zittel Conklin & Westen [83]	Clinician self-report 79 items Assesses CT patterns in a psychotherapeutic setting on a wide range of cognitive, affective and behavioral responses therapists have to their patients. 5-point Likert scale ranging from 1 (not true) to 5 (very true).	The English version revealed an eight-factor structure [88]: - *Overwhelmed/Disorganized* factor indicates a desire to avoid or flee the patient and strong negative feelings, including dread, repulsion, and resentment. - *Helpless/Inadequate* factor describes feelings of inadequacy, incompetence, hopelessness, and anxiety. - *Positive* factor indicates the experience of a positive working alliance and close connection with the patient. - *Special/Overinvolved* factor describes a sense of the patient as special, relative to other patients, or describes ‘soft signs’ of problems in maintaining boundaries, including self-disclosure, ending sessions on time, and feeling guilty, responsible, or overly concerned about the patient. - *Sexualized* factor describes sexual feelings toward the patient or experiences of sexual tension. - *Disengaged* factor describes feeling distracted, withdrawn, annoyed or bored in sessions. - *Parental/Protective* factor describes a wish to protect and nurture the patient in a parental way, above and beyond normal positive feelings toward the patient. - *Criticized/Mistreated* factor describes feelings of being unappreciated, dismissed, or devalued by the patient. The Italian version [89] revealed nine dimensions that are very similar to those of the English version, except for the original Criticized/Mistreated pattern, which seems to be split into the following two factors. - *Hostile/Angry* indicates feelings of anger, hostility, and irritation toward the patient. - *Criticized/Devalued* describes the sense of being criticized, unappreciated, dismissed, or devalued by the patient. The German version [81] identified the following five factors (which largely overlap greatly with those of the English and Italian versions): - *Hostile* - *Positive* - *Disengaged* - *Overwhelmed* - *Encroaching* includes feelings of compassion, sadness, depression, anger at people in patient’s life, “walking on eggshells”, fear of saying anything wrong because the patient could explode.
Therapist Response Questionnaire—Suicide Form (TRQ-SF) Yaseen et al. [90]	Clinician self-report 10 items Assesses clinicians’ responses to suicidal patients after a single encounter. It includes five items derived from the TRQ [83], two items from the WAI [91] and three items developed de novo. 5-point Likert scale ranging from 0 (not at all) to 4 (extremely).	Three subscales have been identified and validated by Barzilay et al. [92] and adopted by Michaud et al. [93]: - *Affiliation* - *Distress* - *Hopefulness*
Vignettes Rating Scale (VRS) Brody & Faber [45]	Clinician self-report 20 items Assesses the extent to which the clinician imagined that actually working with the patient described in a clinical vignette would generate a variety of feelings and reactions. 5-point Likert scale, with response options ranging from 1 (much less than usual) to 5 (much more than usual).	Three different aspects of countertransference: - *Positive countertransference* includes feelings of liking the patient, engagement, compassion, empathy, nurturance, helpfulness, gratification, and challenge. - *Negative countertransference* includes feelings of boredom, anxiety, irritation, anger, frustration, hopelessness, and depression. - *Countertransference-related behavior* includes the tendency or temptation to give advice, to think about the patient during leisure time, to refer the patient elsewhere, to let sessions run over time, and let the patient know you value and/or like them.

## Data Availability

Not applicable.
